# Clinical Audit of screening Latent TB, Hepatitis-C, and Occult Hepatitis-B in Rheumatoid arthritis’ patients starting biologic or targeted synthetic DMARDS

**DOI:** 10.12669/pjms.41.6.11427

**Published:** 2025-06

**Authors:** Hafiz Yasir Qamar, M. Ahmed Saeed, M. Rafaqat Hameed, Maryam Aamer, Umbreen Arshad, Amar Lal

**Affiliations:** 1 Hafiz Yasir Qamar, Central Park Medical College, Rheumatology, Lahore, Pakistan; 2 M. Ahmed Saeed, Central Park Medical College, Rheumatology, Lahore, Pakistan. National Hospital & Medical Center, Rheumatology, Lahore, Pakistan. Arthritis Care Foundation, Lahore, Arthritis Care Center, Lahore, Pakistan; 3 M. Rafaqat Hameed, Central Park Medical College, Rheumatology, Lahore, Pakistan. National Hospital & Medical Center, Rheumatology, Lahore, Pakistan. Arthritis Care Foundation, Lahore, Arthritis Care Center, Lahore, Pakistan; 4 Maryam Aamer, Central Park Medical College, Rheumatology, Lahore, Pakistan; 5 Umbreen Arshad, Central Park Medical College, Rheumatology, Lahore, Pakistan; 6 Amar Lal, Central Park Medical College, Rheumatology, Lahore, Pakistan

**Keywords:** Biologic drugs, Hepatitis, Rheumatoid arthritis, TB, tsDMARDS

## Abstract

**Objective:**

To highlight the importance of pre-treatment screening in Rheumatoid arthritis’ patients starting biologic or targeted synthetic DMARDS.

**Methods:**

This retrospective multicentric clinical audit was conducted at the Department of Rheumatology, Central Park Teaching Hospital and affiliated Rheumatology Clinics in Lahore, Pakistan. All patients enrolled gave an informed consent, meeting the ACR/EULAR 2010 criteria for RA, aged 17 years or above, and initiating b/ tsDMARDS between November, 2019 and March, 2024. Data for this clinical audit was retrieved from biologic and targeted synthetic DMARDS registry of consortium of four centers including two academic centers. Data was retrospectively reviewed and analyzed using SPSS version 26.

**Results:**

The cohort comprised 353 patients, predominantly female 280 (79.3%) with a mean age of 47.55 years. Before starting treatment, patients were screened for latent TB, HCV, and HBV. Compliance with latent TB documentation was 98%, with 20.2% of patients testing positive and receiving appropriate treatment, without any reactivation of TB during treatment. Screening for occult HBV revealed 14.7% tested positive for antibodies, with 5.1% showing active infection, all of whom were treated. For HCV, 9.6% tested positive, but none required further treatment as per HCV RNA.

**Conclusion:**

This clinical audit emphasizes the need of thorough screening protocols with early detection and management of latent infections to optimize treatment outcomes in RA particularly in regions with specific infectious disease burdens like Pakistan. Our documentation of compliance with pre-screening was high but there remains room for improvement. We aim to clinical audit for reactivation of these infections also in future.

## INTRODUCTION

Biological medicines are agents that target one immunological or genetic mediator specifically that contributes to the development of a disease.^1^ In rheumatology, these medications represent significant therapeutic options, especially for individuals who are resistant to conventional disease modifying drugs. These medications are Anti-TNF, B-cell depleting agents,IL-1, IL-6 inhibitors, CTLA-4 and JAK inhibitors. These biological drugs, which alleviate symptoms and limit disease development, have transformed the treatment of many immune-mediated inflammatory conditions, including rheumatic diseases. However, due to their immunosuppressive effects, they can have a variety of adverse effects, including latent TB reactivation, HBV and HCV reactivation, and demyelinating disorders.^2^

According to the EULAR 2022 recommendations for screening and prophylaxis of chronic infections in adults with rheumatic diseases, screening for chronic HBV, HCV, and latent TB is advised in individuals prior to starting b/ts DMARDs. Screening for latent tuberculosis should follow national and/or international guidelines. Due to inadequate screening, this can lead to a significant annual reactivation rate of these infections patients on b/ts DMARDs.^3^

Rheumatoid arthritis (RA) is the common autoimmune rheumatic disease and because of the increased incidence of these chronic infections in our part of the world^4^ in which majority are unidentified or unaware of their occult infections status, the patients of RA starting biologics need meticulous pre-infectious screening as per the guidelines mentioned to provide good rheumatology care taking into consideration the burden of these infections. This study primarily aimed to evaluate the adherence to screening protocols for latent TB, HBV, and HCV in RA patients in a Pakistani cohort. Secondary objective was to find out the proportion of patients with these infections starting biologic or targeted synthetic DMARDS in RA.

## METHODS

This retrospective, multicentric clinical audit was conducted at the Department of Rheumatology, Central Park Teaching Hospital, and affiliated rheumatology clinics in Lahore, Pakistan. The study involved convenient sampling of patients with RA according to the 2010 ACR/EULAR criteria. Data were collected from the biologic and targeted synthetic DMARDs registry of a consortium of four centers, two of which are academic centers. All those patients who initiated treatment with b/ts DMARDs between November, 2019 and March, 2024 were included in this audit. Patients with prior exposure to b/tsDMARDs were excluded from the study. A written informed consent was obtained from all patients.

### Ethical Approval:

The study received approval from the participating centers’ institutional ethical review boards (Ref. No.: CPMC/IRB-No/1459A, Date: April 10, 2024), and all data were handled in accordance with the Declaration of Helsinki’s ethical criteria.

### Data collection:

Data was extracted retrospectively from electronic health records. Patient demographic characteristics, including age, gender, and disease duration, were recorded. Serologies of patients, including RF and anti-CCP antibodies, were retrieved. Information on prior and post-initiation DMARDs was extracted, along with pre-treatment screening results for latent TB, occult HBV, and HCV. A history of previous TB, HBV, or HCV infection, as well as any treatment for these conditions, was also noted.

Interferon-gamma release assays (IGRAs) and chest X-rays were used to screen for latent TB. The positive results were handled in compliance with national guidelines. HBsAg, Anti-HBc, and Anti-HBs were tested serologically as part of latent HBV screening. If Anti-HBc was detected, prophylactic treatment was started irrespective of HBV DNA status by Hepatologist. Anti-HCV antibody tests were performed for hepatitis C screening, and where necessary, HCV RNA PCR was employed to establish active infection.

### Statistical analysis:

Data was analyzed using SPSS version 26. Categorical variables were given as frequencies and percentages, whereas continuous variables were presented as mean and standard deviation. Demographic and clinical characteristics were analyzed using descriptive statistics such as mean, standard deviation, and percentages. Chi-square tests were used to compare proportions between groups, with significance set at p-value < 0.05.

## RESULTS

**Fig.1:**
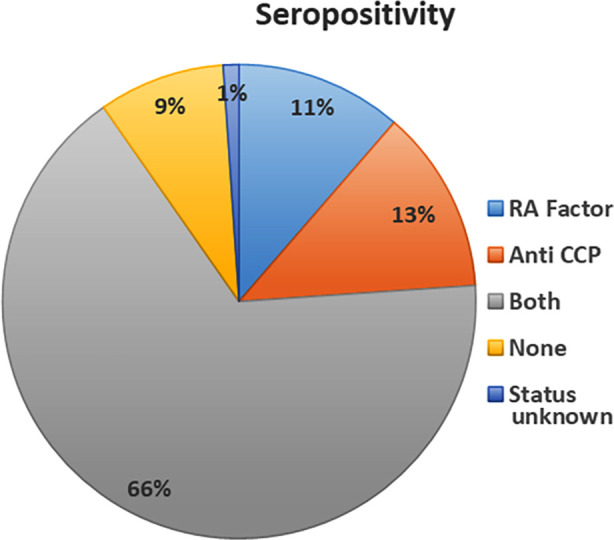
Distribution of Sero-positivity Among Patients Based on RA Factor and Anti-CCP Levels.

A total of 353 patients were included, with 73 males (20.7%) and 280 females (79.3%). The mean age was 47.55 years (SD ± 12.82), ranging from 20 to 76 years. The disease, duration varied from one to 34 years, with a mean of 12.18 years (SD ± 6.13). Most patients had been living with RA for more than 10 years, indicating a chronic disease course. The majority of patients 322 (91%) tested positive for either RA Factor, Anti-CCP or both, while 31 (8.8%) tested negative (Fig.1).

**Fig.2:**
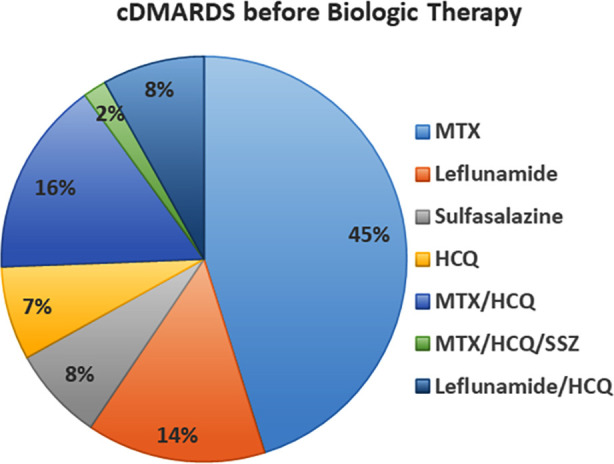
Proportion of cDMARDs Use in Patients Before Starting Biologic Therapy

Before initiating b/tsDMARDs, patients were prescribed various conventional DMARDs (cDMARDs). Methotrexate (MTX) was the most commonly used cDMARD (n=145, 41.1%). Other cDMARDs included leflunomide (n=46, 13.0%), sulfasalazine (n=24, 6.8%), and hydroxychloroquine (HCQ) (n=24, 6.8%). Combination therapies were also prevalent, with MTX and HCQ being the most common combination (n=50, 14.2%). A small number of patients were not on any cDMARD at the time of initiating biologic therapy (n = 14, 4.0%) (Table-I) (Fig.2).

**Table-I:** cDMARDs in Patients Before Starting Biologic Therapy

DMARDS on starting biologics					
		Frequency	Percent	Valid Percent	Cumulative Percent
Valid	MTX	145	41.1	41.1	41.1
	Leflunamide	46	13.0	13.0	54.1
	Sulfasalazine	24	6.8	6.8	60.9
	HCQ	24	6.8	6.8	67.7
	MTX/HCQ	50	14.2	14.2	81.9
	MTX/HCQ/SSZ	6	1.7	1.7	83.6
	Leflunamide/HCQ	26	7.4	7.4	90.9
	Other combination	18	5.1	5.1	96.0
	None	14	4.0	4.0	100.0
	Total	353	100.0	100.0	

**Fig.3:**
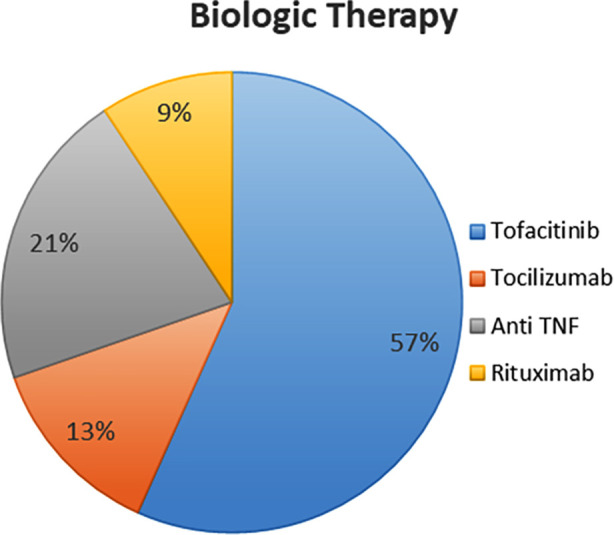
Distribution of Biologic Treatments Initiated

Among the biologic treatments initiated, Tofacitinib was the most commonly used, (n = 200, 56.7%). Anti-TNF agents were used in 74 patients (21.0%), tocilizumab in 46 patients (13.0%), and rituximab in 33 patients (9.3%) m (Fig.3).

Latent TB screening using IGRA and X-ray chest was done in 346 patients (98.0%). A small proportion of patients 6 (1.7%) had a previous history of Tuberculosis (TB), 3 (0.85%) had active TB on X-ray chest and later MTB DNA testing of sputum and other 3 (0.85%) had old changes. While the majority 340 (96.3%) had no previous or active TB. Compliance to documentation for Latent TB was 98%. Patients who tested positive for latent TB were 70 (20.2%). They were treated as per National TB guidelines. Six patients (2%) were not documented but they were on treatment, (Table-II).

**Table-II:** Latent TB Screening, Results, and Treatment Summary.

Parameter	Frequency	Percent (%)	Valid Percent (%)	Cumulative Percent (%)
***Previous TB***				
Yes	6	1.7	1.7	1.7
No	347	98.3	98.3	100.0
***Latent TB Screening (IGRA)***				
Done	346	98.0	98.0	98.0
Not available	7	2.0	2.0	100.0
***IGRA***				
Positive	70	19.8	20.2	20.2
Negative	270	78.5	79.8	100.0
Missing	6	1.7		
***Chest X-ray***				
Normal	340	98.3	98.3	98.3
Old changes	3	0.85	0.85	100.0
***Latent TB Treatment***				
Yes	70	19.8	19.8	19.3
No	270	80.7	80.7	100.0

All 353 patients (100.0%) were screened for Occult HBV, with 52 (14.7%) testing positive for Anti HBc Total/IgG antibody, and 18 (5.1%) patients additionally tested positive for HBsAg. All 52 individuals were preemptively treated for Hepatitis B without monitoring HBV DNA levels. (Table-III). All patients (100%) were screened for HCV using anti-HCV antibody tests. Thirty-four patients (9.6%) tested positive for anti-HCV antibodies, but subsequent HCV RNA testing revealed that none had active infection (all HCV RNA-negative). Therefore, no patients required antiviral treatment for HCV at the time of initiating b/ts DMARDs therapy, (Table-IV).

**Table-III:** Screening and Treatment of Occult Hepatitis B (HBV).

Screening & Treatment of Occult Hepatitis B (HBV)	Frequency (n)	Percent (%)	Valid Percent (%)	Cumulative Percent (%)
***Occult Hepatitis B Screening***				
Done	353	100.0	100.0	100.0
***Occult Hepatitis B Screening Result (Anti HBc IgG/Total)***				
Positive	52	14.7	14.7	14.7
Negative	301	85.3	85.3	100.0
***HBsAg***				
Positive	18	5.1	5.1	5.1
Negative	335	94.9	94.9	100
***Hepatitis B Treatment***				
Yes	52	14.7	14.7	14.7
No	301	85.3	85.3	100.0

**Table-IV:** Screening and Treatment of Occult Hepatitis C (HCV)

Hepatitis C Screening	Frequency	Percent	Valid Percent	Cumulative Percent
Hep C Screening Performed	353	100.0	100.0	100.0
Hep C Screening Result				
Positive	34	9.6	9.6	9.6
Negative	319	90.4	90.4	100.0
HCV RNA Test Result				
Negative	34	9.6	9.6	9.6
Not Applicable (HCV RNA not required)	319	90.4	90.4	100.0
Hep C Treatment				
Not Treated (No Active Infection)	353	100.0	100.0	100.0

There were no cases of TB or HBV reactivation among patients who received prophylactic therapy during the study period. This demonstrates the value of pre-treatment screening and care in preventing infection reactivation in patients receiving immunosuppressive therapy.

## DISCUSSION

Chronic infections, defined as those that manifest more frequently or more severely in individuals with compromised immune systems, are found in the context of AIIRD and are frequently linked to immunosuppressive and immunomodulatory therapies used to treat these conditions.^5^ The findings of this study highlight how crucial it is to screen for latent infections in RA patients prior to starting b/ts DMARDs.

An estimated 1.3 million people die yearly from infections with the HBV and HCVs globally.^6^ According to statistics, there are currently 248 million people with chronic HBV infection, 71.1 million HCV-antibody positives, and 80 million persons with active viraemic infections.^7^,^8^ LMICs, especially in Asia and Africa, face a significant burden of chronic HBV and HCV, yet most infected individuals remain unaware of their condition despite improvements and advancements for treatment. Patients receiving immunosuppressive medication are divided into four risk groups according to their HBV serology results about the possibility of HBV reactivation. Patients who are HBsAg-positive and anti-HBc-positive and receiving rituximab or ofatumumab are classified as very high-risk (reactivation risk > 20%). HBsAg-negative, anti-HBc-positive patients taking rituximab or ofatumumab, as well as those on anthracycline derivatives and high-dose prednisolone, are classified as high-risk (reactivation risk: 11-20%). Patients classed as medium-risk (reactivation risk: 1-10%) include those who are anti-HBc positive, HBsAg-positive, or HBsAg-negative who are on low-dose prednisolone, T cell inhibitors, or anti-TNF drugs. Patients classified as low-risk (reactivation risk <1%) comprise HBsAg-negative and anti-HBc-positive individuals taking drugs such as methotrexate or azathioprine, as well as short-term corticosteroids or low-dose prednisolone.^1^

This study shows 100% adherence to HBV screening; 14.7% of patients have positive Anti-HBc test results, indicating latent HBV infection or previous exposure. HBsAg tested 5.1% positive of them, a marker of ongoing, chronic infection. Regardless of their HBV DNA levels, treating every patient who tested positive for anti-HBc indicates a cautious approach to controlling possible HBV reactivation. This strategy is in accordance with a number of therapeutic recommendations, such as the 2015 guidelines from the American Gastroenterology Association, which suggest antiviral prophylaxis for patients receiving immunosuppressive medication and having serological evidence of past or currently active HBV infection. While routine oral antiviral prophylaxis is advised for patients at high and intermediate risk, but not for low risk patients. Every patient scheduled for biological treatment needs to have an HBV screening done.^9^

The national RISE registry of US practices shows that fewer than one-third of patients had complete HBV testing. There is enough evidence of pervasive inadequacies in HBV screening prior to immunosuppression.^10^ HCV infection, like HBV, carries a minimal risk of reactivation. In Pakistan, where HCV is still a major public health concern, the 9.6% prevalence of anti-HCV antibodies in this cohort is in line with HCV prevalence rates from previous research. Crucially, no patient with detectable HCV RNA was found in those who tested positive for anti-HCV antibodies, indicating that they had successfully achieved an SVR after prior antiviral therapy.

Acute hepatitis with liver failure, ALT rise, and subclinical viremia are among the manifestations of HCV reactivation. High amounts of intrahepatic HCV-RNA have been found in both immunocompetent and immunocompromised hosts with minimal to no liver damage, suggesting that HCV seems less cytopathic than HBV. As a result, when HCV is reactivated, clinical hepatitis is typically less severe than HBV.^11^ The prevalence of HCV seropositivity is less well-documented. Only six patients (1.7%) out of 344 RA patients in a cross-sectional investigation tested positive for anti-HCV.^12^ Another study involving 220 individuals with skin psoriasis, both with and without arthritis, revealed that 4% of the participants had a current HCV infection.^13^

“Prophylactic” antiviral medication is not now useful for individuals with treated or cured HCV infection who are going to undergo immunosuppression. Moreover, anti-viral prophylaxis is not a practical strategy due to the extended course of treatment needed for rheumatic diseases. Peg-interferon and ribavirin were the primary therapies for HCV previously. Interferon use needs to be cautious because it can aggravate some rheumatic conditions and psychological disorders. In individuals with ongoing HCV infection, co-administration of immunosuppression and anti-viral medication seems to be a suitable method.^14^-^16^

None of the HCV antibody-positive patients in this study required additional therapy because the HCV RNA PCR findings were all negative. When initiating long-term immunosuppressive medication, persons with resolved HCV infection—that is, those who test negative for HCV RNA but nonetheless have positive anti-HCV antibodies—should still be vigilantly monitored for any evidence of liver damage or delayed reactivation which is necessary to ensure patient safety even though HCV reactivation is less common than HBV.

In a country like Pakistan, where TB remains a significant public health concern, rigorous pre-treatment screening for latent TB is crucial, particularly in patients with autoimmune conditions like RA, where immunosuppression is an inherent part of the therapeutic strategy. There is no gold standard for identifying LTBI, despite the possibility that active TB can be identified by a patient’s medical history, clinical examination, chest radiography, and bacteriological examination. Divergent recommendations have been made about the use of screening instruments such the IGRAs and TST.^17^ The two tests were compared in a number of studies, which confirmed inconsistent results.^18^ The current statistics confirm that IGRAs are superior to the TST in identifying persons with a prior history of BCG vaccination. In fact, IGRAs are highly helpful in areas where BCG was given during infancy or in situations involving frequent booster shots.^19^ Therefore, this study uses IGRA for screening; a 20.2% rate of latent TB indicates that the screening method is useful for identifying people who are at risk. The high compliance (98%) with latent TB screening highlights the adherence to established guidelines and demonstrates the clinical commitment to ensuring patient safety before initiating immunosuppressive therapies such as b/tsDMARDs.

The ACR recommends that a patient with LTBI be directed to a specialist without disclosing the recommended course of treatment.^20^ Guidelines suggest biologics should be started one to two months after LTBI prophylaxis, with anti-TNF-α treatment if started one month after prophylaxis significantly reduce TB reactivation in RA patients tested positive for LTBI, providing support for the claim.^21^

The most significant aspect of official guidelines is to analyses their results, i.e., whether or not rheumatologists actually implement the recommendations, and finally, how the guidelines alter the course of TB in patients taking DMARDs. Reactivation of LTBI in RA patients is uncommon these days, unless there is a lack of compliance with the guidelines. In Spain, patients with RA treated with anti-TNF-α medicines experienced a significant decrease in their chance of getting active tuberculosis after national recommendations were released. The number of active tuberculosis cases decreased from 32 to two, and the incidence rate per 100,000 people declined from 523 to 112.^22^ Maintaining strict adherence to official criteria, as in RCTs, enhances the treatment’s effectiveness in preventing Mtb infection reactivation. Golimumab treatment in phase III clinical trials resulted in 317 latent tuberculosis infections out of the 2,210 individuals after complete TB screening, but none developed active tuberculosis after completing course of isoniazid therapy.^23^

Notably, in our study, all patients who tested positive for latent TB received treatment in accordance with the National TB Guidelines, and no cases of TB reactivation were reported during the follow-up period. The absence of TB reactivation in this study demonstrates the cohort’s adherence to guidelines. This data is especially relevant since it indicates the efficiency of latent TB treatment in preventing reactivation when b/ts DMARDs are administered. Only 2% of patients who had inadequate TB screening documentation while receiving prophylactic treatment suggests that further improvements in record-keeping and follow-up are still required.

### Strength of the study:

This study’s strength is its assessment of Pakistani RA patients’ compliance with latent TB, HBV, and HCV screening guidelines. It complies with national and international standards and provides a thorough overview of current practices. The large sample size of the study guarantees its validity and usefulness in clinical settings. Despite these advantages, several areas still need more research. It also provides epidemiological statistics on the prevalence of latent TB, occult HBV, and HCV in RA patients, highlighting the significance of routine screening and preventive therapy. It doesn’t assess the efficacy of prophylactic strategies or long-term patient outcomes. Cost, accessibility, and physician awareness—factors that impact adherence—are not considered. Additionally, the study does not evaluate liver function outcomes in patients with HBV and HCV, and there is little information available regarding the relative cost-effectiveness of risk-based versus universal screening strategies in settings with limited resources.

### Limitations

While the study provides valuable insights, there are certain limitations that need to be considered. As a retrospective audit, it is inherently dependent on the accuracy and completeness of existing clinical records, which may introduce biases due to missing data or incomplete documentation. The analysis did not stratify outcomes by the specific b/ts DMARDs used, which could offer differing risks for infection reactivation, limiting a more tailored risk assessment. The study’s focus on short-term outcomes restricts understanding of delayed reactivation events, as long-term follow-up data were not available. Additionally, its geographic limitation to a single region may limit generalizability to populations with different epidemiological profiles. The study also does not account for patient-related factors like adherence to therapy and socioeconomic status, which could influence infection risks. Evaluating these aspects in future prospective studies would provide a more comprehensive risk assessment.

## CONCLUSION

In conclusion, this audit signifies the importance of comprehensive infection screening before initiating b/ts DMARDs in RA patients. Effective implementation of screening protocols for HBV, HCV, and TB, coupled with appropriate prophylactic measures, plays a critical role in preventing infection reactivation ensuring patient safety during immunosuppressive therapy. The high compliance rates for screening, particularly 98% for latent TB and 100% for HBV and HCV, indicate the effectiveness of current protocols in detecting potential risks before initiating treatment. Enhanced documentation practices and continued research into tailored management strategies will further optimize the care of patients with AIIRD undergoing immunosuppressive therapy.

### Authors Contribution:

**HYQ, MAS & MRH:** Concept, design, Manuscript writing and data analyses.

**HYQ, MAS, MRH**, **MA** **& AL:** Literature review.Critical analysis.

**MAS, MRH & UA:** Data collection. Critical review.

**HYQ:** Responsible and accountable for the accuracy of the study.

All authors have read and approved the final version.
